# Myocardial Injury and Postoperative Hypotension in the Recovery Room Are Not Correlated: A Retrospective Cohort Study

**DOI:** 10.3390/jcm14197083

**Published:** 2025-10-07

**Authors:** Thijmen J. J. de Ruiter, Robert Jan Stolker, Sanne E. Hoeks, Felix van Lier

**Affiliations:** Department of Anesthesiology, Erasmus MC, 3015 GD Rotterdam, The Netherlands; t.deruiter@erasmusmc.nl (T.J.J.d.R.); r.stolker@erasmusmc.nl (R.J.S.); f.vanlier@erasmusmc.nl (F.v.L.)

**Keywords:** myocardial injury, hypotension, non-cardiac surgery, anesthesia

## Abstract

**Background:** Hypotension is common during and after surgery and is known to be associated with myocardial injury. This study investigated whether hypotension in the recovery room after non-cardiac surgery is associated with myocardial injury. **Methods:** In this retrospective cohort study, data were used from a clinical registry of patients aged 60 years or older undergoing intermediate-to-high-risk non-cardiac surgery. Patients admitted to the Intensive Care Unit or the high-dependency ward were excluded. Hypotension was characterized by the time-weighted average (TWA) below 75 mmHg mean arterial pressure (MAP) measured in the recovery room after surgery. Patients were divided into quartiles of increasing hypotension exposure, i.e., increasing TWA. The primary outcome was myocardial injury, defined as peak high-sensitivity troponin T over 50 ng/L within the first 3 postoperative days. **Results:** In total, 2660 patients were analyzed. Hypotension in the recovery room was observed in 1119 (43.7%) of the patients while myocardial injury occurred in 198 (7.7%) patients. There was no association between increasing exposures of hypotension and myocardial injury (lowest TWA vs. highest TWA quartile: 7.9% vs. 9.0%, *p* = 0.65). Furthermore, there was no association between hypotension and 30-day mortality (highest vs. lowest quartile: 1.5% vs. 1.5%, *p* = 0.82). However, an association was noted at 1 year (9.8% vs. 14.7%, *p* = 0.02). **Conclusions:** Hypotension in the recovery room after moderate-to-high-risk non-cardiac surgery is not associated with the development of myocardial injury.

## 1. Introduction

Postoperative myocardial infarction is a severe cardiovascular complication after non-cardiac surgery [1,2]. However, diagnosing it can be challenging due to the sedative and analgesic effects of general anesthesia and postoperative analgesics, which mask the subjective symptoms—one of the three criteria for formalizing the diagnosis [3]. Diagnosis of myocardial infarction necessitates objective necrosis of myocardial tissue, referred to as myocardial injury: an elevation in cardiac troponin to at least above the 99th percentile [3]. Myocardial injury is just as relevant as myocardial infarction with respect to prognosis in the perioperative setting [4]. Multiple studies have shown that myocardial injury is associated with cardiovascular and non-vascular morbidity and mortality [5,6,7]. For these reasons, the focus in recent research has shifted from myocardial infarction to myocardial injury. The occurrence of myocardial injury is up to 54% in intermediate-to-high-risk non-cardiac surgery, depending on the definition used [4,5].

Several studies investigated how to reduce the incidence and consequences of perioperative myocardial infarction [8,9]. Two major trials could not show any mortality benefit from the heart rate-lowering drugs clonidine and metoprolol, most probably as a result of increased intra- and postoperative hypotension resulting in an increase in cerebrovascular events [8,9,10]. Since then, there has been a heightened focus on the risk associated with hypotension. The POISE I trial even notes that clinically significant hypotension is the largest population attributable contributor to death [9]. In a recent narrative review, the importance of both prevention of hypotension and prevention of tachycardia was emphasized [11].

Hypotension is a common occurrence during and after surgery [12,13]. The vasodilative and cardiodepressive properties of the anesthetics used, the compression of major vascular structures, inflammation and relative or absolute hypovolemia are major contributors to this phenomenon [14]. Although hypotension per se does not equate to hypoperfusion and ischemia, there have been multiple studies describing worse outcomes after exposure to hypotension during and after surgery [6,8,9,10,15,16,17,18]. Due to the physiology of coronary perfusion and comparatively high oxygen extraction of myocardial tissue, hypotension may be especially relevant in the development of myocardial injury.

Postoperative hypotension and its relation with myocardial injury has been previously studied at different time points and with different definitions [18,19,20,21]. A recent study showed that postoperative hypotension in the first 24 h after non-cardiac surgery, and not intraoperative hypotension, was associated with myocardial injury in patients admitted to a high-dependency ward after surgery [17]. It is still unknown what blood pressure level, in the phases after the surgery is critical and whether the relationship between hypotension and myocardial injury is also present in the presumably more low-risk patients admitted to regular wards after short-term recovery in a dedicated recovery room. We set out to bridge this gap in knowledge.

The research aim of this study is to investigate whether hypotension during the recovery period after intermediate-to-high-risk non-cardiac surgery in non-high-dependency patients is associated with postoperative myocardial injury.

## 2. Materials and Methods

### 2.1. Ethics

The institutional research board of the Erasmus MC, Rotterdam, The Netherlands (METC Erasmus MC) approved this study as exempt from the Medical Research Involving Human Subjects Act (WMO) (Decision MEC-2014-659, 6 January 2015) and exempted the study from the need for informed consent, in accordance with the local directive for retrospective studies.

This study was not deposited in a study registry and complies with the Helsinki declaration on research ethics. This report follows the Strengthening The Reporting of Observational Studies in Epidemiology (STROBE) criteria for observational studies.

### 2.2. Study Design

The study population is based on a previously described single-center observational cohort [5,17,18]. Data were extracted from a clinical routine troponin registry of non-cardiac surgery patients at the Erasmus Medical Center, Rotterdam, The Netherlands. All patients aged 60 years and older undergoing intermediate-to-high-risk non-cardiac surgery were included. The 2014 ESC Guidelines on Non-Cardiac Surgery: Cardiovascular Assessment and Management were followed concerning the operative risk of surgery [22]. A minimum expected postoperative hospital stay of 24 h was required for inclusion. Data was included between July 2012 and July 2017. This dictated the number of included patients and consequently the sample size.

All patients received standard care. After surgery, patients were transferred to the intensive care unit, the high-dependency ward, or the recovery room, from which the patient was subsequently discharged to the general ward of their respective surgical departments. The determination of the intended postoperative destination for elective surgery was established during the outpatient pre-operative anesthesiology clinic visit, considering the type of surgery and patient characteristics. This study focuses on the population assigned to the recovery room and regular ward for postoperative care. Patients admitted to the intensive care unit or high-dependency ward were excluded from the analysis as they went directly to their respective postoperative location bypassing the recovery room.

The recovery room in the Erasmus MC hospital consisted of a single large multi-patient room. In the recovery room, each patient was monitored with at least a five-lead ECG, continuous pulse-oximetric saturation monitoring and a non-invasive blood pressure cuff. Patients could be mechanically ventilated and receive vasopressor support for a short period, if indicated. Patients were discharged from the recovery room to the regular ward based on the Aldrete score system. This system rates the recovery from general anesthesia on 5 domains on a 0 to 2-point scale: motor activity, respiratory rate, blood pressure, alertness and oxygen saturation [23]. Patients required a total score of 9 to be discharged.

### 2.3. Data Collection

Patient characteristics were obtained manually from the pre-operative medical records of the patient in the digital hospital information system (Chipsoft Hix, Amsterdam, The Netherlands). This was supplemented by exporting data from the institution’s electronic medical records concerning prescribed medication, vital signs at the outpatient clinic and on the ward, and administrative data concerning the admission of the patient. Based on the patient history data and prescription data exported, the Revised Cardiac Risk index (RCRI) was calculated [24]. Postoperative high-sensitive troponin-T was routinely collected as part of standard care protocols on postoperative days 1, 2 and 3, provided the patient remained admitted to the hospital. The Cobas e602 Troponin T hs STAT assay (Roche Diagnostics, Mannheim, Germany) was used for troponin tests.

### 2.4. Hemodynamic Data

Hemodynamic and respiratory measurements in the pre-operative holding area, the operating room and the recovery room were automatically recorded within a hospital anesthesia information management system as an integral part of routine clinical care. This system contains information on administered medication, fluid balance including blood loss, and other surgical variables. The times for the start of anesthesia, the start of surgery, the end of surgery and, where applicable, the start and end of recovery were manually recorded into the same system. Patients without hemodynamic data during the recovery period were excluded as their exposure to hypotension could not be ascertained.

Blood pressure measurements consisted of non-invasive oscillometric blood pressure supplemented by invasive arterial blood pressure, if available. Non-invasive blood pressure was recorded at the programmed measurement interval. The default measuring interval was set to 5 min in the recovery room and 3 min in the operation room. Invasive blood pressure measurements were recorded at a 5 min interval and at each moment of non-invasive measurement. The interval was allowed to be individualized by the care team. In accordance with a previously published algorithm, the hemodynamic data was cleaned, excluding the following: a systolic blood pressure lower than 20 mmHg or greater than 300 mmHg; a diastolic blood pressure either less than 20 mmHg or greater than 200 mmHg; a diastolic pressure greater than the systolic pressure and a pulse pressure smaller than 20 mmHg [25]. If both invasive and non-invasive data were present, invasive measurements were used. The mean arterial pressure (MAP) was calculated from the systolic and diastolic blood pressure. After the filter was applied, the blood pressure was interpolated linearly to a 1 min interval.

### 2.5. Hypotension Thresholds

Hypotension in the recovery ward was characterized using the time-weighted average under threshold (TWA) of the MAP. The TWA was calculated by taking the area under the threshold and dividing this by the time spent in the recovery ward. For intraoperative hypotension, two other characterizations, apart from TWA, were also reported: time under threshold (TuT) and area under threshold (AuT). Based on previous publications, we selected a threshold of 75 mmHg MAP as the primary cutoff for postoperative hypotension [17]. In a secondary analysis, two lower thresholds, 70- and 65-mmHg, were selected for analyses.

### 2.6. Outcome Measures

Myocardial injury was defined as an elevation in the Hs-TnT exceeding 50 ng/L within the first three postoperative days, constituting the primary outcome. This definition is in accordance with previously published papers based on this cohort and our institutional policy [5,6,17,18]. Patients without any Hs-TnT values were excluded from the analysis.

For all patients with elevated troponin levels, the presence or absence of postoperative myocardial infarction was determined using the criteria according to the third universal definition, which was the current definition at the start of inclusion [26].

Secondary outcomes included postoperative blood pressure recordings on the regular ward, as well as 30-day and 1-year mortality. Blood pressure measurements were ascertained at least once per 8 h nursing shift, though local department protocols and clinical judgment could shorten the interval. All available measurements were used to calculate the mean MAP per day, per patient. Survival status was completed in all patients by means of the institution’s medical records or was ascertained by inquiry from civil registries.

### 2.7. Statistical Analysis

Continuous variables are presented as the mean and SD or as the median and interquartile range (IQR) when data were not normally distributed. Visual inspection of Q-Q plots was, together with the Shapiro–Wilk test, used to check for normality. Categorical variables are presented as numbers and percentages. Exposure to hypotension was characterized based on the TWA under MAP 75 divided in quartiles.

One-way ANOVA and the Chi-squared-test were used for comparison of baseline characteristics and outcomes across the exposure quartiles. For non-parametric tests, the Kruskal–Wallis test was used. A Kaplan–Meier survival analysis including LogRank testing was performed with censoring at 365 days after surgery.

We performed a multivariate linear analysis and logistic regression analyses to correct for age, gender (ref = female), pre-operative blood pressure (MAP in mmHg), blood loss during surgery (milliliters), length of surgery (minutes) and RCRI (nominal: 0 (ref), 1, 2, 3, >3). Any cases with 1 or more missing covariates were excluded. Due to the skewness of the hs-TnT value’s, the analysis was performed using the log-transformation with a small-constant correction to prevent log(0). R-statistics version 4.2.1 was used for all statistical analyses. A *p* < 0.05 was selected as a threshold for significance. Bonferroni correction for multiple testing was applied with respect to the 5 groups, resulting in an adjusted threshold of <0.01 as the threshold for significance.

## 3. Results

**Figure 1:**
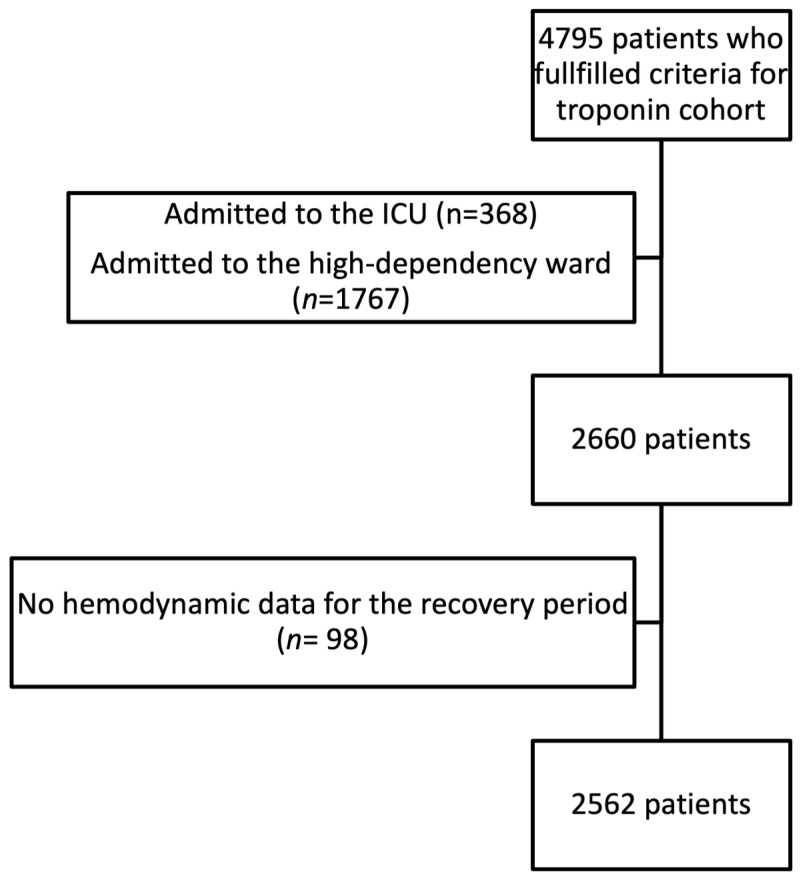
Flow diagram depicting inclusion and exclusion of patients in the cohort. ICU—intensive care unit.

In total, 4795 patients were included in the cohort (Figure 1), of whom 2660 were admitted to the recovery room. An additional 98 patients were excluded from the analysis due to missing hemodynamic data during the recovery period. Consequently, 2562 patients were available for the current analysis.

### 3.1. Baseline Characteristics

Baseline characteristics per categorized TWA hypotension quartiles are presented in Table 1. The median age in the population was 69.2 years [IQR 64.8–74.8]. The majority of patients underwent a procedure under general anesthesia (95.8%). Vascular procedures (38,9%) and urologic/gynecologic procedures (20.9%) were the most prevalent types of surgery. Of all surgeries, 5.2% were classified as ‘emergency’. The Revised Cardiac Risk Index showed no significant relationship with the exposure to postoperative hypotension (*p* = 0.227). Differences were found among the quartiles of exposure for age, sex, duration of surgery, the history of coronary disease and renal disease, and the pre-operative MAP.

### 3.2. Hemodynamic Exposure

Intraoperative blood pressures per postoperative hypotension quartile are presented in Table 2. Patients who had lower blood pressures during surgery often had more profound hypotension in the recovery room after surgery as well. Intraoperative hemodynamic exposure was significantly different for the different quartiles of exposure to postoperative hypotension. The quartile with the most profound postoperative hypotension was also exposed to the most hypotension during surgery, independently of the intraoperative threshold or the quantification method used.

The median time on the recovery was 68 min [IQR 33–120] with a median number of 13 blood pressure measurements [IQR 3–25] recorded (Table 3). In 897 (35.1%) patients, at least one invasive blood pressure measurement was recorded during the recovery period. Hypotension (defined as MAP < 75 mm Hg) was present in 1119 (43.7%) patients. Hypotensive patients were grouped in four quartiles based on their respective TWA under MAP 75 mm Hg. This corresponded to a per-group median of the per-patient mean MAP during the recovery period ranging from 86 mmHg (IQR 82–92) in quartile 1 (least hypotension) to 70 mmHg (IQR 67–75) in the 4th quartile (most hypotension).

In our supplementary analyses for MAP < 70 mmHg and MAP < 65 mmHg, the incidence of hypotension was evidently lower (respectively, 32.2% and 21.9% (Supplementary Material Tables S1 and S2).

### 3.3. Primary Outcome

Overall, postoperative myocardial injury was present in 191 (7.5%) patients (Table 3). There was no association observed between hypotension ansd the incidence of myocardial injury (*p* = 0.19). Among patients with hypotension, myocardial injury ranged from 6.1% to 10.4% without a discernible pattern. In the secondary analyses involving additional thresholds of 70 mmHg and 65 mmHg, no significant relation was identified either (Supplementary Material Tables S1 and S2). The overall incidence of myocardial infarction after surgery was 1,8% (*n* = 46) with no significant differences across exposure quartiles (*p* = 0.29).

Multivariate regression analyses showed no significant effect of postoperative hypotension exposure on hs-TnT value’s, myocardial injury or infarction. (see Table 4).

### 3.4. Secondary Outcomes

The mean MAP in the ward on the first 3 postoperative days differed significantly between groups (Table 3). Upon pairwise analysis, no clear pattern could be discerned among the quartiles.

**Figure 2:**
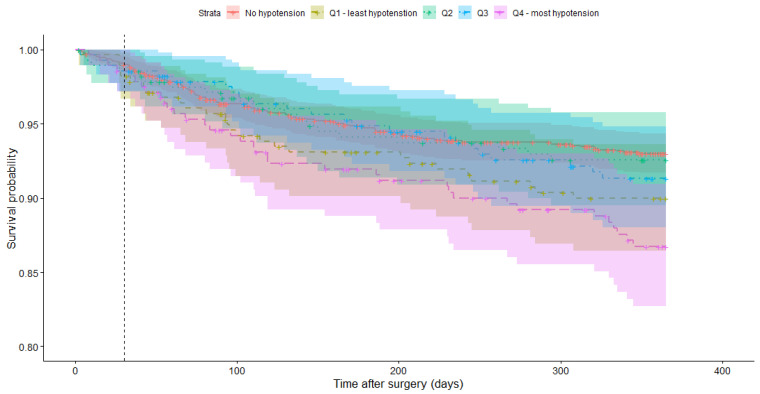
Kaplan–Meier curve depicting the probability of survival for each of the 5 groups. The dashed line represents 30 days after surgery.

Overall mortality was 1.3% at 30 days and 8,9% at 1 year postoperatively. There was no difference in the incidence of mortality at 30 days (1,0% vs. 1.4%. *p* = 0.85), although a significant difference was found for 1-year mortality (7.6% vs. 14.5%, *p* = 0.009) (Table 3). Further analysis using the Kaplan–Meier analysis and log-rank test showed a significantly lower probability of survival at 1 year (0.93 vs. 0.86, Chi^2^ = 12.7, df = 4, *p* = 0.013, see Figure 2).

## 4. Discussion

This retrospective cohort study investigated whether hypotension in the recovery room after intermediate or high-risk non-cardiac surgery was associated with myocardial injury. The current study does not demonstrate a relationship between recovery room hypotension, expressed as a TWA below 75 mmHg, and the occurrence of myocardial injury in the first three days after surgery, contrary to previous findings in the literature [17,20]. This conclusion remains unchanged after correcting for pre-operative and postoperative confounders using multivariable regression analysis. Furthermore, no association was seen between hypotension and 30-day mortality or myocardial infarction, nor was an effect noted at the lower thresholds of MAP 70 mmHg or 65 mmHg for myocardial injury.

Several previous studies showed that hypotension after non-cardiac surgery was associated with myocardial injury [17,20]. The current study differs from these papers with respect to the studied population, severity of the hypotensive exposure and the extent of postoperative care provided. A comparable study focused on a population admitted to a high-dependency ward and found a notably higher overall incidence of myocardial injury with increasing exposure to postoperative hypotension [17]. We purposefully selected a more low-risk population for our study, as we hypothesized that this effect would also be present in low-risk patients, and we deemed the exclusion of these patients as a limitation to the aforementioned study. We intended to bridge this gap in the knowledge on this important subject.

A second study, by Roshanov et al., investigated the relationship between different degrees of coronary artery sclerosis and perioperative hypotension in relation to cardiovascular death or nonfatal myocardial infarction, which entails elevated troponin [20]. All patients received a CT-coronary angiogram before surgery and the scans were rated on a range from normal to extensive obstructive disease. In this study an association between hypotension, both intra- and postoperatively, and the primary outcome was noted. This study did not observe an interaction between the severity of coronary obstruction, hypotension and postoperative myocardial infarction or mortality [20]. The difference in outcome between the current study and the study by Roshanov may be attributed to differences in how hypotension was defined. There, postoperative hypotension was defined as a systolic blood pressure < 90 mmHg with any intervention initiated to counter it. A post hoc analysis showed that, in our population, only the three most exposed quartiles had more than 1 min of exposure to systolic < 90 mmHg.

Recently, a systematic review on the effects of both hypo- and hypertension in both cardiac and non-cardiac surgery was published [21]. Despite using different definitions on what constitutes hypotension, multiple included studies described an increased mortality after longer or more profound exposure to hypotension. Concerning the cardiovascular risk of hypotension specifically, this review did highlight an increased risk for “myocardial injury, myocardial damage and acute myocardial infarction” connected to perioperative hypotension. The authors focused on the prevention of hypotension during and directly after surgery but did not separately investigate these phenomena.

A large cohort study by Khanna et al., published before the aforementioned review but not included in it, did specifically test for the interaction between intraoperative hypotension and postoperative hypotension and the incidence of major adverse cardiac and cerebrovascular events (MACCEs) [27]. They found that postoperative hypotension was not associated with MACCE for patients without intraoperative hypotension. Similarly, in patients with intraoperative hypotension, postoperative hypotension was not linked to MACCE, except at an extreme threshold of 55 mmHg. The authors did describe an effect of hypotension at all tested thresholds on secondary outcomes including acute kidney injury and hospital readmission.

The effects of early hypotension during recovery are supported by a recent study where the association between intra- and postoperative hypotension and ward hypotension was evaluated [28]. Intraoperative blood pressure predicted hypotension on the ward poorly, whereas early postoperative blood pressures showed a modest association with patients experiencing hypotension on the ward. Our study results confirm that patients who have hypotension in the recovery room have lower blood pressures on the ward on the day of surgery, though it should be noted that these differences are statistically but not clinically significant in our cohort.

A recent randomized control study, the POISE III trial, investigated multiple hypotheses in a 2 × 2-methodology including the hypothesis that a hypotension prevention strategy (which included a intraoperative goal for the MAP > 80 mmHg) would be superior to a hypertension prevention strategy (MAP > 60 mmHg intraoperatively) for the prevention of myocardial injury after surgery [29]. This study concluded that the proposed hypotension-avoidance strategy did not differ in the incidence of a combined range of cardiovascular complications including myocardial injury, despite having a significant effect on both intraoperative and postoperative blood pressure [29]. This further affirms the findings of our cohort study where hypotension until 65 mmHg did not significantly increase the risk of myocardial injury. A post hoc analysis of exposure to MAP < 60 mmHg during the recovery room stay further suggested that even lower thresholds may not increase the risk of myocardial injury. However, the decreasing sample size and consequent reduction in statistical power should be considered (see Supplemental Table S3).

Our study focusses on the effect of postoperative hypotension in the recovery room. We recognize that the pre-operative status of the patient and intraoperative course, including blood loss and duration of surgery, for example, might have a more profound effect than or confound the effect of postoperative hypotension. We included a cursory table of intraoperative hemodynamic data to underline the association between intraoperative and postoperative hemodynamics. In an attempt to reduce the effect of pre-operative and intra-operative covariates, we performed a multivariate analysis. Correction for multiple variables did not change the fact that there was a lack of association between postoperative hypotension and myocardial injury or any of the other primary endpoints. It should be noted, however, that, due to the low incidence and consequent low amount of events/variable for myocardial injury, and even more so for myocardial infarction, this analysis might be underpowered. For this analysis, we elected not to correct for vasopressor use during the recovery stay as we deemed a single-variable approach unsuitable to capture the complex effect of vasopressors on organ perfusion. Furthermore, any single variable is unable to capture the complex temporal relationship between changes in vasopressor dossing and measured hypotension.

The current study has several limitations. First, this retrospective study is limited by the exposure to hypotension in the cohort. Using the selected threshold for hypotension, MAP < 75 mmHg, only 46% of the patients had at least one documented hypotensive measurement. For lower thresholds, the incidence decreased further, down to just 23% for MAP < 65. Furthermore, we opted to characterize hypotension by the TWA under the threshold of 75 mmHg. A choice was made in favor of this characterization rather than time-under-threshold or the area-under-threshold based on the theoretical consideration that a non-time corrected measurement might amplify the impact of a patient with an unfavorable postoperative course who consequently would have had a prolonged stay in the recovery room. Furthermore, the threshold of MAP < 75 mmHg is debatable, as there is no universal consensus on what constitutes hypotension [30]. A recent study utilizing machine learning algorithms published in Anesthesia and Analgesia even suggested that diastolic blood pressure is a better predictor than the MAP [31]. Moreover, the definition used for our primary outcome does not correspond to current day definitions proposed by the VISION trial [32] and the 4th Universal Definition of Myocardial Infarction [3], which have a lower cutoff value for defining myocardial injury. We executed our study based on the definition agreed upon at the inception of the cohort, which might limit its external validity to current day practice. Any case of myocardial injury found in our study satisfies both the biochemical criteria of the VISION definition and the criteria in accordance with the 4th Universal Definition. It should be noted that for the VISION definition, there should also be a clinical judgment as to the likelihood of the different causes of troponin elevation and the VISION trial looked for biochemical changes for up to 30 days postoperatively. As we cannot retrospectively perform this clinical assessment nor obtain more hs-TnT samples, we cannot retrospectively apply the VISION trial definition to our cohort. The 4th Universal definition requires a rise-and-fall pattern to ascertain the acuteness of the myocardial injury. For most of the patients in our cohort, only the required three daily Troponin levels were obtained, and no pre-operative hs-TnT was available as that was only introduced as standard care in the 2022 ESC guidelines on the prevention of cardiac complications from non-cardiac surgery [33].

Secondly, there was a risk for selection bias, as the most high-risk patients were preselected for a postoperative stay on the high-dependency ward or ICU. This was reflected in the lower overall incidence of myocardial injury for the recovery population, and consequently, this limited the power of the current study.

A third limitation exists in the treatment bias. The blood pressure treatment goals were not documented in this cohort. Considering the lower prevalence of cardiovascular and renal disease in the more hypotensive quartiles, it is possible that these patients were assessed as having a higher risk for injury due to hypotension. Consequently, higher blood pressure goals might have been set and prolonged vasopressor support was actively utilized in the recovery room. This could have led to the selection of relatively low-risk patients for the more hypotensive quartiles, thereby reducing the likelihood of detecting myocardial injury. Furthermore, the retrospective design inherently limits the ability to establish causality between postoperative hypotension and myocardial injury. While a prospective, randomized methodology could potentially address this issue, we consider it unfeasible due to significant ethical challenges of allowing hypotension despite evidence of worse outcomes [15,16] and the substantial resources required for the required size of such a study. Additionally, the single-center setting may limit the generalizability of the findings to other institutions or patient populations. Finally, the reliance on mean arterial pressure as the sole measure of hypotension may not capture the full complexity of hemodynamic changes in the postoperative period. Even though research is still inconclusive about the additive value on clinical outcomes, more multi-factorial modalities like the Hypotension Prediction Index might be beneficial for predicting hypotension and consequently perhaps even for predicting myocardial injury [34].

The recent literature has investigated multiple potential pre-operative predictors for myocardial injury and other cardiopulmonary complications [35,36,37]. Among these, we reported the RCRI. Although these studies have not demonstrated improved prediction by adding the American Association of Anesthesiologists—Physical Status (ASA—PS) score, its near-universal adoption in clinical practice might have facilitated the clinical interpretation of our findings. However, the ASA—PS score was not routinely extracted for our cohort and therefore could not be reported.

In our secondary outcomes, the difference in 1-year mortality was notable. Although the 30-day mortality showed no association with hypotension, the 1-year mortality did show a significant association. These findings, we hypothesize, might be explained by seeing postoperative hypotension as a marker for the susceptibility of the cardiovascular system, rather than a direct cause of mortality. This hypothesis is supported by our Kaplan–Meier curve showing a consistently steeper declining probability for survival, rather than a sudden sharp decrease one would expect if the perioperative hypotension directly led to myocardial infarction or other acute ischemic events.

Based on the current study, a previously described blood pressure threshold (MAP < 75 mmHg) linked to an increased risk of postoperative myocardial injury in high-dependency ward patients does not predict myocardial injury in general ward patients after intermediate to high-risk non-cardiac surgery. Additional analyses do not support the lower thresholds of 70 mmHg or 65 mmHg, although this conclusion is drawn from smaller samples. Further research should focus on why hypotension is predictive of myocardial injury in high-risk patients but not in lower-risk patients, as well as on the predictive value of preoperative biomarkers, hypotension during surgery and the interaction of vasopressors with regard to the correlation of hypotension and postoperative myocardial injury

## 5. Conclusions

In patients undergoing intermediate-to-high-risk non-cardiac surgery, the severity of hypotension in the recovery room, expressed as the TWA under MAP 75 mmHg, is not associated with myocardial injury.

## Figures and Tables

**Table 1:** Baseline characteristics stratified to quartiles of the time-weighted average (TWA) under MAP 75 mmHg.

	Overall	No Hypotension	Q1—Least Hypotension	Q2	Q3	Q4—Most Hypotension	*p*
**N**	2562	1443	280	280	280	279	
**Postoperative MAP TWA under 75 mmHg (mmHg)**	0.00 [0.00–1.12]	0.00 [0.00–0.00]	0.13 [0.05–0.25]	0.87 [0.58–1.20]	2.60 [2.06–3.39]	6.87 [5.43–9.22]	<0.001 *
**Demographic and surgery**							
**age (years)**	69 [65–75]	69 [64–74]	70 [66–76]	70 [66–76]	69 [65–74]	70 [65–74]	0.005 *
**Sex = male**	1496 (58.4%)	818 (56.7%)	188 (67.1%)	178 (63.6%)	160 (57.1%)	152 (54.5%)	0.004
**emergency = no**	2430 (94.8%)	1356 (94.0%)	267 (95.4%)	266 (95.0%)	272 (97.1%)	269 (96.4%)	0.144
**Duration of surgery (minutes)**	133 [96–185]	125 [90–167]	139 [102–188]	150 [110–195]	151 [108–217]	157 [109–225]	<0.001 *
**Blood loss (mL)**	100 [0–300]	100 [0–250]	118 [10–300]	100 [0–300]	150 [108–217]	150 [20–400]	<0.001 *
**Surgical specialty**							
**General surgery**	317 (12.4%)	146 (10.1%)	35 (12.5%)	33 (11.8%)	45 (16.1%)	58 (20.8%)	
**Neurosurgery**	223 (8.7%)	159 (11.0%)	26 (9.3%)	10 (3.6%)	13 (4.6%)	15 (5.4%)	
**Orthopedic surgery**	333 (13.0%)	216 (15.0%)	25 (8.9%)	40 (14.3%)	30 (10.7%)	22 (7.9%)	
**Urology/Gynaecology**	536 (20.9%)	259 (17.9%)	56 (20.0%)	72 (25.7%)	75 (26.8%)	74 (26.5%)	
**Vascular surgery**	997 (38.9%)	556 (38.5%)	120 (42.9%)	115 (41.1%)	106 (37.9%)	100 (35.8%)	
**Other**	156 (6.1%)	107 (7.4%)	18 (6.4%)	10 (3.6%)	11 (3.9%)	10 (3.6%)	
**Cardiovascular risk factors**							
**Revised Cardiac Risk Index**							0.227
**0**	1327 (51.8%)	749 (51.9%)	135 (48.2%)	140 (50.0%)	149 (53.2%)	154 (55.2%)	
**1**	784 (30.6%)	462 (32.0%)	89 (31.8%)	73 (26.1%)	78 (27.9%)	82 (29.4%)	
**2**	323 (12.6%)	168 (11.6%)	40 (14.3%)	45 (16.1%)	39 (13.9%)	31 (11.1%)	
**3**	107 (4.2%)	53 (3.7%)	13 (4.6%)	17 (6.1%)	12 (4.3%)	12 (4.3%)	
**>3**	21 (0.8%)	11 (0.8%)	3 (1.1%)	5 (1.8%)	2 (0.7%)	0 (0.0%)	
**Pre-operative MAP (mmHg)**	99 [91–106]	100 [92–107]	98 [91–105]	97 [91–105]	98 [90–105]	95 [88–105]	<0.001 *
**Hypertension**	1387 (54.1%)	797 (55.2%)	162 (57.9%)	151 (53.9%)	150 (53.6%)	127 (45.5%)	0.031
**Myocardial infarction**	245 (9.6%)	115 (8.0%)	36 (12.9%)	33 (11.8%)	28 (10.0%)	33 (11.8%)	0.026
**Coronary artery disease**	371 (14.5%)	182 (12.6%)	48 (17.1%)	57 (20.4%)	47 (16.8%)	37 (13.3%)	0.005
**Congestive heart failure**	88 (3.4%)	46 (3.2%)	13 (4.6%)	13 (4.6%)	7 (2.5%)	9 (3.2%)	0.477
**Diabetes mellitus**							0.660
**Insulin-dependent**	196 (7.7%)	111 (7.7%)	22 (7.9%)	23 (8.2%)	24 (8.6%)	16 (5.7%)	
**Insulin-independent**	338 (13.2%)	192 (13.3%)	46 (16.4%)	34 (12.1%)	33 (11.8%)	33 (11.8%)	
**Cerebrovascular accident**	395 (15.4%)	202 (14.0%)	42 (15.0%)	52 (18.6%)	50 (17.9%)	49 (17.6%)	0.155
**Renal disease**	280 (10.9%)	192 (13.3%)	34 (12.1%)	31 (11.1%)	16 (5.7%)	7 (2.5%)	<0.001
**COPD**	301 (11.7%)	165 (11.4%)	31 (11.1%)	41 (14.6%)	31 (11.1%)	33 (11.8%)	0.618
**Peripheral arterial disease**	356 (13.9%)	201 (13.9%)	44 (15.7%)	39 (13.9%)	38 (13.6%)	34 (12.2%)	0.830
**Pre-operative medication**							
**Calcium Chanel blockers**	571 (22.3%)	339 (23.5%)	64 (22.9%)	57 (20.4%)	59 (21.1%)	52 (18.6%)	0.373
**Angiotensin-II inhibitors**	461 (18.0%)	254 (17.6%)	54 (19.3%)	50 (17.9%)	60 (21.4%)	43 (15.4%)	0.410
**ACE-inhibitors**	545 (21.3%)	292 (20.2%)	58 (20.7%)	67 (23.9%)	60 (21.4%)	68 (24.4%)	0.439
**Betablockers**	867 (33.8%)	495 (34.3%)	105 (37.5%)	92 (32.9%)	91 (32.5%)	84 (30.1%)	0.420
**Diuretics**	626 (24.4%)	360 (24.9%)	76 (27.1%)	60 (21.4%)	70 (25.0%)	60 (21.5%)	0.401
**Statins**	1105 (43.1%)	605 (41.9%)	134 (47.9%)	134 (47.9%)	123 (43.9%)	109 (39.1%)	0.095
**Aspirin**	761 (29.7%)	415 (28.8%)	87 (31.1%)	94 (33.6%)	89 (31.8%)	76 (27.2%)	0.371
**Oral anticoagulants**	244 (9.5%)	146 (10.1%)	28 (10.0%)	24 (8.6%)	24 (8.6%)	22 (7.9%)	0.713
**Nitrates**	104 (4.1%)	52 (3.6%)	12 (4.3%)	16 (5.7%)	10 (3.6%)	14 (5.0%)	0.462

*—According to the non-parametric Kruskal–Wallis test. MAP—mean arterial pressure, TWA—Time-weighted average, COPD—Chronic obstructive pulmonary disease, ACE—Angiotensine converting enzyme.

**Table 2:** Intra-operative hemodynamic data stratified to quartiles of the Time-Weighted Average (TWA) under MAP 75 mmHg during recovery.

	Overall	No Hypotension	Q1—Least Hypotension	Q2	Q3	Q4—Most Hypotension	*p*
**N**	2540	1430	278	279	276	277	
**Postoperative MAP TWA under 75 mmHg (mmHg)**	0.00 [0.00–1.12]	0.00 [0.00–0.00]	0.13 [0.05–0.25]	0.87 [0.58–1.20]	2.60 [2.06–3.39]	6.87 [5.43–9.22]	<0.001 *
**Intraoperative**							
**MAP AuT 65 mmHg (mmHg** **·** **min.)**	107 (178)	85 (141)	113 (181)	89 (132)	144 (245)	196 (256)	<0.001
**MAP AuT 70 mmHg (mmHg** **·** **min.)**	243 (336)	196 (278)	253 (332)	208 (252)	318 (421)	441 (478)	<0.001
**MAP AuT 75 mmHg (mmHg** **·** **min.)**	483 (563)	392 (481)	501 (556)	437 (438)	623 (660)	840 (768)	<0.001
**MAP TuT 65 mmHg. (min.)**	18 (27)	15 (22)	19 (25)	15 (20)	24 (31)	34 (39)	<0.001
**MAP TuT 70 mmHg (min.)**	36 (42)	29 (36)	37 (41)	33 (34)	45 (46)	62 (56)	<0.001
**MAP TuT 75 mmHg (min.)**	59 (57)	48 (51)	61 (56)	58 (50)	75 (61)	94 (69)	<0.001
**Duration of operation (min.)**	145 (74)	133 (66)	152 (77)	155 (69)	167 (88)	169 (83)	<0.001
**MAP TwA under 65 mmHg. (mmHg)**	0.62 (1.00)	0.54 (0.94)	0.62 (0.94)	0.50 (0.82)	0.75 (1.24)	0.97 (1.18)	<0.001
**MAP TwA under 70 mmHg (mmHg)**	1.36 (1.77)	1.21 (1.68)	1.38 (1.70)	1.13 (1.46)	1.63 (2.04)	2.14 (2.07)	<0.001
**MAP TwA under 75 mmHg (mmHg)**	2.66 (2.81)	2.35 (2.69)	2.70 (2.75)	2.33 (2.32)	3.13 (3.04)	4.02 (3.16)	<0.001

*—According to the non-parametric Kruskal–Wallis test, MAP—mean arterial pressure, TWA—Time-weighted average, AuT—Area under threshold, TuT—Time under threshold.

**Table 3:** Primary and secondary outcomes stratified to quartiles of the Time-Weighted Average (TWA) under MAP 75 mmHg.

	Overall	No Hypotension	Q1—Least Hypotension	Q2	Q3	Q4—Most Hypotension	*p*
**N**	2562	1443	280	280	280	279	
**Postoperative MAP TWA under 75 mmHg (mmHg)**	0.00 [0.00–1.12]	0.00 [0.00–0.00]	0.13 [0.05–0.25]	0.87 [0.58–1.20]	2.60 [2.06–3.39]	6.87 [5.43–9.22]	<0.001 *
	Primary outcomes						
**Hs-TnT (ng/L)**	14 [9–23]	15 [9–23]	15 [10–24]	15 [10–23]	14 [9–21]	13 [9–23]	0.319 *
**Postoperative myocardial injury**	191 (7.5%)	98 (6.8%)	22 (7.9%)	29 (10.4%)	17 (6.1%)	25 (9.0%)	0.192
**Postoperative myocardial infarction**	46 (1.8%)	20 (1.4%)	8 (2.9%)	5 (1.8%)	5 (1.8%)	8 (2.9%)	0.289
	Secondary outcomes						
**Mean MAP during recovery (mmHg)**	89 [80–99]	97 [90–104]	86 [82–92]	81 [78–87]	77 [75–82]	70 [67–74]	<0.001 *
**Duration of recovery stay (minutes)**	68 [33–120]	43 [28–84]	93 [61–137]	100 [59–149]	122 [69–170]	117 [72–165]	<0.001 *
**Median MAP at regular ward, day 0 (mmHg)**	99 [91–108]	101 [92–109]	98 [92–107]	99 [91–108]	99 [89–108]	96 [87–104]	<0.001 *
**Median MAP at regular ward, day 1 (mmHg)**	95 [88–103]	96 [89–105]	93 [87–99]	95 [89–107]	96 [87–103]	92 [80–98]	0.080 *
**Median MAP at regular ward, day 2 (mmHg)**	98 [90–106]	98 [92–107]	97 [90–106]	94 [89–99]	99 [91–104]	95 [80–101]	0.004 *
**Median MAP at regular ward, day 3 (mmHg)**	96 [85–104]	98 [88–107]	90 [84–100]	97 [89–101]	95 [89–101]	88 [80–100]	0.029 *
**Mortality at 30 days**	32 (1.3%)	15 (1.0%)	5 (1.8%)	4 (1.4%)	4 (1.4%)	4 (1.4%)	0.845
**Mortality at 1 year**	203 (8.9%)	98 (7.6%)	27 (10.8%)	20 (7.8%)	23 (9.4%)	35 (14.5%)	0.009

*—According to the non-parametric Kruskal–Wallis test; MAP—mean arterial pressure; TWA—time-weighted average; Hs-TnT—high-sensitivity troponin T.

**Table 4:** Uni- and multivariate analysis of the primary outcomes.

	High-Sensitivity Troponine T ^§^									
	Univariate Linear Regression Analysis					Multivariate Linear Regression Analysis *				
	Estimate		95% CI		*p*-Value	Estimate		95% CI		*p*-Value
Intercept	2.722		[2.668–2.774]		<0.001	−0.279		[−0.855–0.298]		0.343
TWA under MAP 75, Q1	0.110		[−0.021–0.241]		0.100	−0.010		[−0.144–0.125]		0.886
TWA under MAP 75, Q2	0.105		[−0.026–0.236]		0.115	−0.019		[−0.155–0.088]		0.787
TWA under MAP 75, Q3	−0.042		[−0.173–0.089]		0.529	−0.070		[−0.214–0.055]		0.248
TWA under MAP 75, Q4	−0.033		[−0.164–0.098]		0.623	−0.049		[−0.186–0.088]		0.483
	**Myocardial injury**									
	Univariate binomial analysis					Multivariate binomial analysis *				
	Estimate	95% CI	Exp (Est.)	95% CI	*p*-Value	Estimate	95% CI	Exp (est.)	95% CI	*p*-Value
Intercept	−2.619	[−2.824–−2.414]	0.073	[0.0594–0.089]	<0.001	−7.429	[−9.856–−5.001]	0.001	[0.000–0.007]	<0.001
TWA under MAP 75, Q1	0.157	[−0.324–0.639]	1.170	[0.7233–1.894]	0.522	−0.010	[−0.566–0.546]	0.990	[0.568–1.726]	0.9718
TWA under MAP 75, Q2	0.461	[0.025–0.897]	1.586	[1.0257- 2.452]	0.038	0.214	[−0.300–0.727]	1.238	[0.741–2.069]	0.4148
TWA under MAP 75, Q3	−0.120	[−0.651–0.412]	0.887	[0.5213 −1.510]	0.659	−0.060	[−0.648–0.529]	0.942	[0.523–1.697]	0.8429
TWA under MAP 75, Q4	0.301	[−0.159–0.760]	1.350	[0.8535–2.138]	0.199	0.480	[−0.041–1.000]	1.615	[0.960–2.718]	0.0709
	**Myocardial infarction**									
	Univariate binomial analysis					Multivariate binomial analysis *				
	Estimate	95% CI	Exp (Est.)	95% CI	*p*-Value	Estimate	95% CI	Exp (est.)	95% CI	*p*-Value
Intercept	−4.265	[−4.706–−3.824]	0.014	[0.009–0.022]	<0.001	−7.158	[−11.595–−2.720]	<0.001	[0.000–0.066]	0.002
TWA under MAP 75, Q1	0.738	[−0.092–1.569]	2.093	[0.912–4.800]	0.081	0.552	[−0.355–1.459]	1.737	[0.701–4.304]	0.233
TWA under MAP 75, Q2	0.258	[−0.731–1.246]	1.294	[0.481–3.476]	0.610	0.199	[−0.821–1.218]	1.220	[0.440–3.382]	0.702
TWA under MAP 75, Q3	0.258	[−0.731–1.246]	1.294	[0.481–3.476]	0.610	0.317	[−0.694–1.328]	1.373	[0.500–3.774]	0.539
TWA under MAP 75, Q4	0.742	[−0.088–1.572]	2.100	[0.916–4.818]	0.080	0.760	[−0.157–1.677]	2.138	[0.855–5.349]	0.104

^§^—Log(hs-TnT + 0.001). *—Corrected for: age, gender, duration of surgery, blood loss, pre-operative MAP and RCRI.

## Data Availability

Data is available on request due to legal restrictions based on the European General Data Protection Regulation.

## Supplementary Materials

The following supporting information can be downloaded at https://www.mdpi.com/article/10.3390/jcm14197083/s1, Table S1. Primary and secondary outcomes stratified to quartiles of the time-weighted average (TWA) under MAP 65 mmHg; Table S2. Primary and secondary outcomes stratified to quartiles of the time-weighted average (TWA) under MAP 70 mmHg; Table S3. Post hoc analyses: primary and secondary outcomes stratified to quartiles of the time-weighted average (TWA) under MAP 60 mmHg.

## Abbreviations

The following abbreviations are used in this manuscript:

ACE	Angiotensin-converting enzyme
ASA—PS	American Association of Anesthesiologists—Physical Status
COPD	Chronic obstructive pulmonary disease
Hs-TnT	High-sensitivity troponin T
ICU	Intensive care unit
IQR	Interquartile range
MACCE	Major adverse cardiac and cerebrovascular events
MAP	Mean arterial pressure
RCRI	Revised cardiac risk Index
SD	Standard deviation
STROBE	Strengthening The Reporting of Observational Studies in Epidemiology
TWA	Time-weighted average
TuT	Time under threshold
AuT	Area under threshold
