# Supplementary material for: Myocardial Injury and Postoperative Hypotension in the Recovery Room Are Not Correlated: A Retrospective Cohort Study
Source: J Clin Med. 2025 Oct 7;14(19):7083. doi: 10.3390/jcm14197083 (PMC12524847; doi:10.3390/jcm14197083)
Supplement: Supplementary file 1 [file jcm-14-07083-s001.zip › Supplementary Material.pdf]

Supplementary Table S1. ***Primary and secondary outcomes stratified to quartiles of the Time-Weighted Average (TWA) under MAP 65 mmHg.***

	Overall	No Hypotension	Q1 – Least hypotension	Q2	Q3	Q4 – Most hypotension	p
N	2562	2001	141	140	140	140	
Post-operative MAP TWA under 65 mmHg (mmHg)	0.00 [0.00, 0.00]	0.00 [0.00, 0.00]	0.04 [0.02, 0.07]	0.24 [0.17, 0.31]	0.70 [0.55, 0.93]	2.56 [1.80, 4.09]	<0.001[a]
Primary outcomes							
Hs-TnT (ng/l)	14 [9, 23]	15 [9, 23]	14 [9, 29]	15 [10, 21]	14 [10, 23]	15 [9, 25]	0.964[a]
Post-operative myocardial injury (%)	191 (7.5)	148 (7.2)	13 (9.6)	11 (8.1)	10 (7.4)	16 (11.9)	0.328
Post-operative myocardial infarction (%)	46 (1.8)	33 (1.6)	5 (3.7)	1 (0.7)	3 (2.2)	4 (3.0)	0.268
Secundary outcomes							
Mean MAP during recovery (mmHg)	89 [80, 99]	92 [85, 102]	80 [76, 85]	77 [73, 82]	76 [72, 81]	68 [64, 73]	<0.001[a]
Median MAP at regular ward, day 0 (mmHg)	99 [91, 108]	100 [92, 108]	97 [89, 108]	99 [90, 107]	99 [89, 107]	97 [58,103.]	0.005[a]
Median MAP at regular ward, day 1 (mmHg)	95 [87, 103]	95 [88, 104]	94 [90, 102]	96 [91, 110]	92 [81, 99]	94 [82, 98]	0.486[a]

	Overall	No Hypotension	Q1 – Least hypotension	Q2	Q3	Q4 – Most hypotension	p
Median MAP at regular ward, day 2 (mmHg)	98 [90, 106]	98 [90, 107]	97 [90, 102]	99 [95, 103]	94 [88, 105]	97 [82, 101]	0.502[a]
Median MAP at regular ward, day 3 (mmHg)	96[85, 104]	96[86, 105]	97 [88, 101]	101 [97, 102]	87 [84, 92]	89 [83, 100]	0.212[a]
Mortality at 30 days (%)	33 (1.3)	22 (1.1)	2 (1.5)	3 (2.2)	4 (3.0)	2 (1.5)	0.310
Mortality at 1 year (%)	205 (8.9)	144 (7.9)	13 (11.2)	14 (11.6)	16 (13.6)	18 (14.8)	0.015

[a] - According to the non-parametric Kruskal-Wallis test. MAP – mean arterial pressure, TWA – Time-weighted average, Hs-TnT – High-sensitivity Troponine-T.

Supplementary Table S2. ***Primary and secondary outcomes stratified to quartiles of the Time-Weighted Average (TWA) under MAP 70 mmHg.***

	Overall	No Hypotension	Q1 – Least hypotension	Q2	Q3	Q4 – Most hypotension	p
N	2562	1737	207	206	206	206	
Post-operative MAP TWA under 70 mmHg (mmHg)	0.00 [0.00, 0.17]	0.00 [0.00, 0.00]	0.06 [0.03, 0.12]	0.48 [0.32, 0.68]	1.48 [1.05, 1.87]	4.28 [3.17, 6.14]	<0.001*
			Primary outcomes				
Hs-TnT (ng/l)	14.00 [9.00, 23.00]	15.00 [10.00, 23.00]	14.00 [9.00, 23.00]	15.00 [10.00, 24.00]	13.00 [9.00, 21.00]	14.00 [9.00, 24.00]	0.620*
Post-operative myocardial injury (%)	191 (7.5)	119 (6.9)	16 (7.7)	21 (10.2)	14 (6.8)	21 (10.2)	0.235
Post-operative myocardial infarction (%)	46 (1.8)	24 (1.4)	6 (2.9)	4 (1.9)	6 (2.9)	6 (2.9)	0.195
			Secundary outcomes				
Mean MAP during recovery (mmHg)	89 [80, 99]	94 [87, 103]	83 [79, 89]	80 [76, 85]	76 [73, 80]	69 [66, 75]	<0.001*
Median MAP at regular ward, day 0 (mmHg)	99 [91, 108]	100 [92, 109]	100 [92, 107]	97 [89, 108]	100 [90, 108]	96 [87, 104]	0.001*
Median MAP at regular ward, day 1 (mmHg)	95 [88, 103]	96 [88, 104]	94 [88, 107]	96 [89, 102]	95 [84, 107]	93 [80, 98]	0.519*

	Overall	No Hypotension	Q1 – Least hypotension	Q2	Q3	Q4 – Most hypotension	p
Median MAP at regular ward, day 2 (mmHg)	98 [90, 106]	98 [91, 107]	94 [90, 99]	98 [92, 102]	99 [90, 105]	95. [80, 100]	0.053*
Median MAP at regular ward, day 3 (mmHg)	96 [85, 104]	96 [87, 106]	98 [83, 102]	96 [91, 100]	91 [84, 99]	89 [82, 100]	0.341*
Mortality at 30 days (%)	32 (1.3)	18 (1.0)	3 (1.4)	4 (2.0)	5 (2.4)	2 (1.0)	0.410
Mortality at 1 year (%)	203 (8.9)	118 (7.6)	20 (10.7)	18 (10.0)	19 (10.6)	28 (15.2)	0.008

* - According to the non-parametric Kruskal-Wallis test. MAP – mean arterial pressure, TWA – Time-weighted average, Hs-TnT – High-sensitivity Troponine-T.

Supplementary Table S3. ***Post-Hoc analyses: Primary and secondary outcomes stratified to quartiles of the Time-Weighted Average (TWA) under MAP 60 mmHg.***

	Overall	No Hypotension	Q1 – Least hypotension	Q2	Q3	Q4 – Most hypotension	p
N	2562	2236	82	82	81	81	
Post-operative MAP TWA under 60 mmHg (mmHg)	0.00 [0.00, 0.00]	0.00 [0.00, 0.00]	0.02 [0.01, 0.04]	0.13 [0.09, 0.16]	0.37 [0.28, 0.51]	1.09 [0.83, 1.86]	<0.001[a]
			Primary outcomes				
Hs-TnT (ng/l)	14 [9, 23]	14 [9, 23]	15.00 [10, 21]	15 [9, 26]	15 [10, 24]	15 [9, 23]	0.869[a]
Post-operative myocardial injury (%)	191 (7.5)	159 (7.1)	6 (7.3)	8 (9.8)	7 (8.6)	11 (13.6)	0.232
Post-operative myocardial infarction (%)	46 (1.8)	37 (1.7)	1 (1.2)	0 (0.0)	4 (4.9)	4 (4.9)	0.027
			Secundary outcomes				
Mean MAP during recovery (mmHg)	89 [80, 99]	91 [83, 101]	78 [72, 82]	77 [72, 83]	75 [69, 79]	67 [62, 72]	<0.001[a]
Median MAP at regular ward, day 0 (mmHg)	99 [91, 108]	100 [92, 108]	103 [93, 110]	99 [90, 107]	102 [93, 109]	95 [88,101]	0.001[a]
Median MAP at regular ward, day 1 (mmHg)	95 [88, 103]	95 [88, 104]	96 [90, 98]	99 [79, 108]	82 [80, 107]	95 [90, 99]	0.833[a]

	Overall	No Hypotension	Q1 – Least hypotension	Q2	Q3	Q4 – Most hypotension	p
Median MAP at regular ward, day 2 (mmHg)	98 [90, 106]	98 [90, 107]	99 [91, 106]	94 [91, 100]	97 [83, 107]	95 [82, 100]	0.328[a]
Median MAP at regular ward, day 3 (mmHg)	96 [85, 104]	96 [86, 105]	82 [80, 91]	95 [87, 98]	89 [86, 108]	89 [85, 99]	0.22[a]
Mortality at 30 days (%)	32 (1.3)	24 (1.1)	2 (2.4)	3 (3.7)	2 (2.5)	1 (1.2)	0.177
Mortality at 1 year (%)	203 (8.9)	161 (8.1)	7 (9.3)	14 (18.9)	8 (10.8)	13 (18.3)	0.001

[a] - According to the non-parametric Kruskal-Wallis test. MAP – mean arterial pressure, TWA – Time-weighted average, Hs-TnT – High-sensitivity Troponine-T.
